# Electron Ptychographic Diffractive Imaging of Boron Atoms in LaB_6_ Crystals

**DOI:** 10.1038/s41598-017-02778-x

**Published:** 2017-06-06

**Authors:** Peng Wang, Fucai Zhang, Si Gao, Mian Zhang, Angus I. Kirkland

**Affiliations:** 1 National Laboratory of Solid State Microstructures, College of Engineering and Applied Sciences and Collaborative Innovation Center of Advanced Microstructures, Nanjing University, Nanjing, 210093 People’s Republic of China; 2 Department of Electrical and Electronic Engineering, Southern University of Science and Technology, Shenzhen, 518055 China; 3 London Centre for Nanotechnology, London, WC1H 0AH UK; 4 Research Complex at Harwell, Harwell Oxford Campus, Didcot, OX11 0FA UK; 5 Department of Materials, University of Oxford, Parks Road, Oxford, OX1 3PH UK; 6 Electron Physical Sciences Imaging Centre, Diamond Lightsource Ltd., Diamond House, Didcot, OX11 0DE UK

## Abstract

Ptychographic diffractive imaging has the potential for structural determination of materials without the constraints of relatively small, isolated samples required for conventional coherent diffractive imaging. The increased illumination diversity introduced using multiple measurements (overlapped probe positions) also provides higher sensitivity to phase changes in weakly scattering samples. The resolution of a ptychographic reconstruction is ultimately determined by the diffraction limit for the wavelength of the radiation used. However, in practical experiments using electrons either the maximum collection angle of the detector used to record the data or the partial coherence of the source impose lower resolution limits. Nonetheless for medium energy electrons this suggests a potential sub 0.1 nm spatial resolution limit, comparable to that obtained using aberration corrected instruments. However, simultaneous visualization of light and heavier atoms in specimens using ptychography at sub 0.1 nm resolution presents a significant challenge. Here, we demonstrate a ptychographic reconstruction of a LaB_6_ crystal in which light B atoms were clearly resolved together with the heavy La atoms in the reconstructed phase. The technique used is general and can also be applied to non-crystalline and extended crystalline samples. As such it offers an alternative future basis for imaging the atomic structure of materials, particularly those containing low atomic number elements.

## Introduction

Accurately determining the location of low atomic number chemical elements in an extended crystal is important in the engineering of many nanometer-scale structures, including hydrogen in hydrogen storage materials^1^, lithium in lithium-ion batteries^2^ and oxygen in electroceramic materials^3^. Transmission Electron Microscopy (TEM) is an established tool for characterizing the structure and chemistry of a wide range of nano-materials^4–6^ and at intermediate voltages aberration corrected TEM or Scanning TEM (STEM) has achieved sub 0.1 nm resolution^7–9^ with sensitivity to light atoms demonstrated using negative Cs TEM^3^ imaging or annular dark field (ADF)^10^ and annular bright field (ABF) STEM imaging^1^. These are significant achievements but typical corrected image resolutions are still *ca*. 20X poorer than the theoretical diffraction limit at medium voltages^11^ being currently limited by residual uncorrected high order aberrations in the objective lens and ultimately by the magnetic field induced by fluctuating currents in the optical column^12^. Furthermore, for samples containing light atoms, quantitative phase data is highly desirable but images recorded using conventional S/TEM imaging modes are unable to directly measure the complex specimen exit surface wave function. Focal or tilt azimuth series reconstruction of the exit wave function^13–18^ provides quantitative phase information. However, in the focal series geometry the resolution of the reconstruction is limited by the effects of partial spatial and temporal coherence and although reconstruction from a tilt azimuth dataset can recover information beyond the axial information limit its implementation requires high precision optical alignment and image registration^14, 18^. An alternative approach to high resolution phase retrieval is provided by electron coherent diffractive imaging (CDI) as initially reported by Weierstall and co-workers^19^ and subsequently by others^20–22^. This method recovers the complex exit surface wave function from a diffraction pattern using a numerical solution to the phase problem. An important feature of CDI is that it recovers the phase with high sensitivity in the presence of experimental detector noise at the level required for the visualization of light elements^22^. There have previously been several of demonstrations of high resolution electron CDI, although as reported, these have generally only been successful for samples containing isolated objects. In addition, to avoid stagnation of the reconstruction algorithm with consequently slow convergence, these previous reports required a low resolution TEM image^21–23^ to act an initial estimate of the object constraint. There is also a significant challenge to using CDI at atomic resolution arising from the need to record weakly scattered intensity located between intense Bragg diffraction peaks.

**Figure 1:**
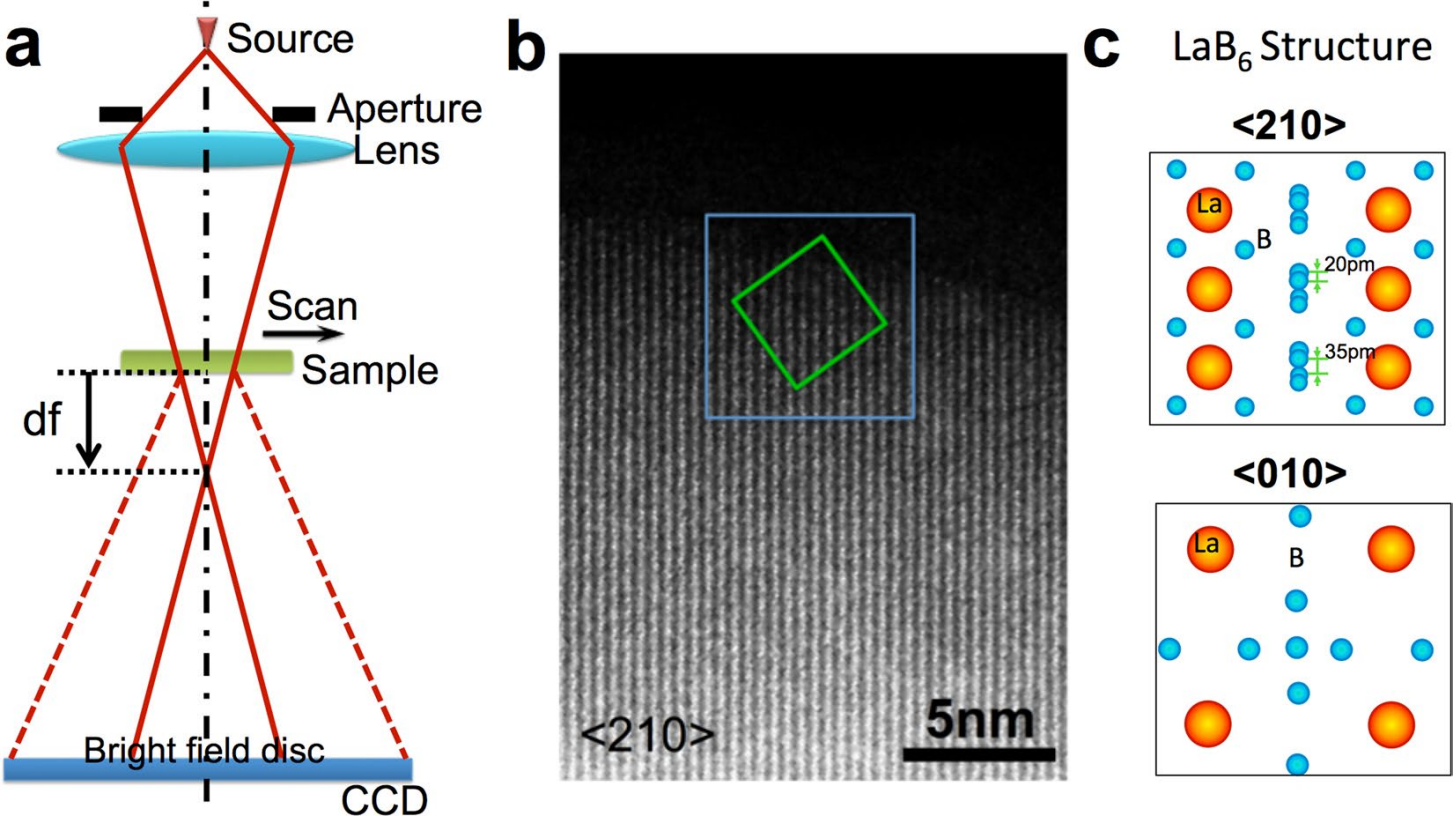
(**a**) Schematic of the experimental configuration used for ptychographic reconstruction. (**b**) HAADF image of a LaB_6_ nanoparticle oriented along a <210> direction. The green box indicates the region where the object function shown in Fig. 2a was restored using the ePIE algorithm. The blue box indicates the region where the HAADF image shown in Fig. 2b was acquired (**c**) Projected atomic models of LaB_6_ along <210> (Top) and <010> (Bottom) directions.

Rodenburg *et al*.^24^ have described ptychographic diffractive imaging, in which a localized illumination probe is moved across a sample while multiple diffraction patterns are recorded from overlapping areas as shown schematically in Fig. 1a. This data acquisition records redundant information and thus overcomes many of the limitations of conventional CDI reconstructions including non-unique solutions and a limited field of view. This data acquisition geometry also enables successful reconstruction without the requirement for *a priori* information about the object^24^. Recovery of local phase changes at atomic resolution can provide structural data directly related to the properties of many important materials^25^ and a suitably sensitive, quantitative phase map can assist structural characterization at the atomic scale^26, 27^. Ptychographic diffractive imaging has been successfully implemented using both light^28^ and X-ray sources^29, 30^. Using electrons^11^, recovery of the object function for gold nanoparticles at a resolution of 0.236 nm has been demonstrated at 30 kV using a convergent electron beam. Putkunz *et al*.^31^ and D’Alfonso *et al*.^32^ have extended the resolution in the recovered exit wavefunction to 0.08 nm using cerium dioxide nanocrystals as a test sample and D’Alfonso *et al*. have further demonstrated the robustness of electron ptychographic reconstructions to varying electron dose^33^. Maiden *et al*.^34^ have shown that a high sensitivity phase signal can be obtained for polystyrene sphere samples at 2.1 nm resolution. However, the full potential of electron ptychography as a tool for imaging weakly scattering samples with high contrast at atomic resolution has not yet been fully realized. If successful, this could offer capabilities in ptychography similar to those available using other techniques, for example, direct imaging of light elements in complex crystal structures, such as cathode materials for lithium-ion batteries^35^. Phase reconstruction may also potentially enable imaging of biological materials containing low atomic number elements without staining, bridging the gap between cellular and molecular biology^36^.

In this paper, we show that using a suitable high resolution scan position-refinement method^37^, electron ptychography can recover structural information for light elements located between heavy elements at atomic resolution and with high phase sensitivity, demonstrated in an experimental reconstruction of a LaB_6_ nanoparticle. The phase data clearly shows both boron (*Z*_B_ = 5) and lanthanum (Z_La_ = 57) atom columns in the LaB_6_ crystal. These initial phase maps of LaB_6_ obtained by ptychography almost reach the quality of phase maps obtained using focal series reconstruction^38^.

## The Ptychographic imaging experiment

The datasets reported here were acquired using a 300 kV (S)TEM Titan G2 cubed 60–300 instrument with a Schottky field emission source. For the datasets described the probe-forming convergence semi-angle was 23 mrad and the corresponding resolution of the instrument is estimated to be 70 pm as measured from images of a GaN <112> crystal in STEM-HAADF images^39^. The electron wavelength used for the data reported here is such that an approximation to a flat Ewald sphere is valid, although we note that this is not generally limiting for this method and can be accommodated by a coordinate transform applied to the recorded diffraction pattern as discussed elsewhere^24, 40^. The sample was located at a distance, *df* above the Gaussian focus point (Fig. 1a). For this study a LaB_6_ nanoparticle supported on a holey carbon film was used, as shown in the high-angle annular dark field (HAADF) STEM image (Fig. 1b). Ptychographic datasets were acquired for two crystal orientations, <210> and <010> with samples located at *df*_<210>_ = 98 nm and *df*_<010>_ = 65 nm, respectively. For both crystal orientations, the incident probe was rastered across a suitable sample region (for example, the area indicated with a green square in Fig. 1b for the case of a <210> orientation) in a grid of either 5 × 5 and 9 × 9 positions, with a nominal step size of 0.5 nm and 0.45 nm, respectively, giving overlaps of ~85% and 80% between adjacent probe positions. Complete (5 × 5 and 9 × 9) arrays of diffraction patterns are shown in Figs S1 and S2 in Supplementary Information (SI). Projected atomic models of the LaB_6_ crystal structure along <210> and <010> directions respectively are shown in Fig. 1c.

In ptychography, an accurate knowledge of the probe translation positions with a precision higher than the resolution of the reconstruction is required. This is particularly challenging for experiments with electrons; firstly because the translations between probe positions are of the order of nm and secondly because both the specimen and probe are subject to positional uncertainty caused by thermal drift, noise in the deflector currents and other instabilities, all of which can be significant during data acquisition^11^ (Fig. S3). The sources of these positional errors, and their inclusion in the ptychographic reconstruction have been addressed using different strategies^37, 41–44^. In this work we have used an automatic position refinement algorithm^37^ that has previously demonstrated sub-pixel accuracy in X-ray and visible light ptychographic experiments. An initial estimate of the probe function *P*_o_(*r*) for the reconstruction is shown in Fig. S4. The sample thickness was estimated as 10 nm with an accuracy of +/−20% over the field of view using the established EELS log-ratio technique (changes in the reconstructed phase due to thickness are shown in Fig. S5 in SI)^45, 46^. The ePIE algorithm^28^ used assumed that the exit wave is a product of the probe function and the object transmission function which is generally satisfied for a relatively thin sample for high energy electrons.

## Phase retrieval of light atoms at atomic resolution

**Figure 2:**
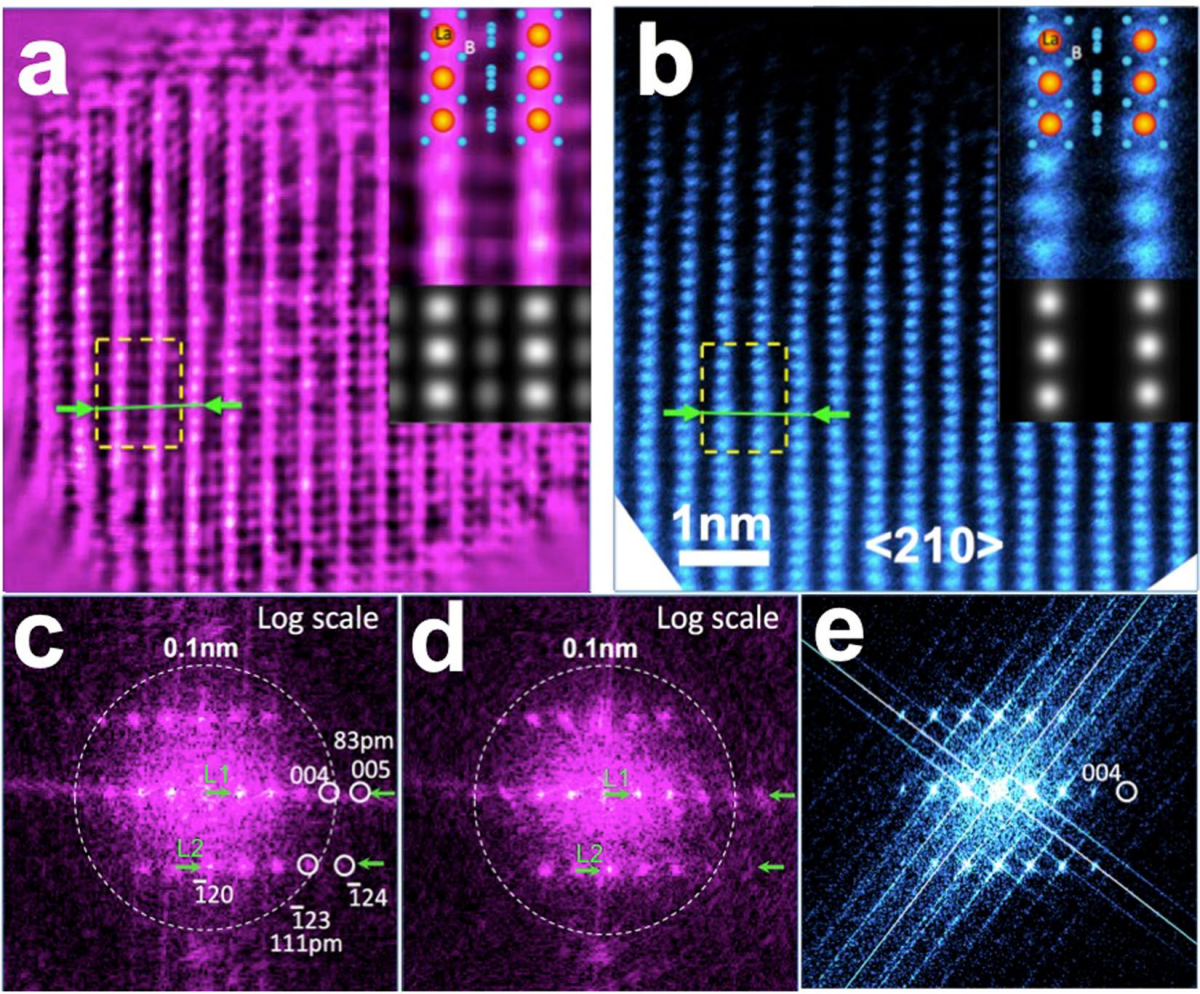
(**a**) Phase of the ptychographic reconstruction corresponding to the green boxed region in Fig. 1b. (**b**) HAADF image from the blue box region in Fig. 1b. (**c**) and (**d**) Power spectra of the reconstructed phase and the complex object wave displayed on a logarithmic intensity scale, respectively. (**e**) Power spectrum of the HAADF image also displayed on a logarithmic intensity scale. Circles indicate (004), $(\bar{1}24)\,\,$and (005) reflections of the LaB_6_ lattice corresponding to spacings of 104 pm, 91 pm and 83 pm. The dotted circle indicates a 100 pm spatial resolution limit. Insets to (**a**) and (**b**). Upper: Enlarged phase of the ptychographic reconstruction and HAADF image extracted from the regions indicated by the yellow dashed squares overlaid with a projected atomic model of the LaB_6_ structure along a <210> direction. Lower: corresponding multislice simulations.

**Figure 3:**
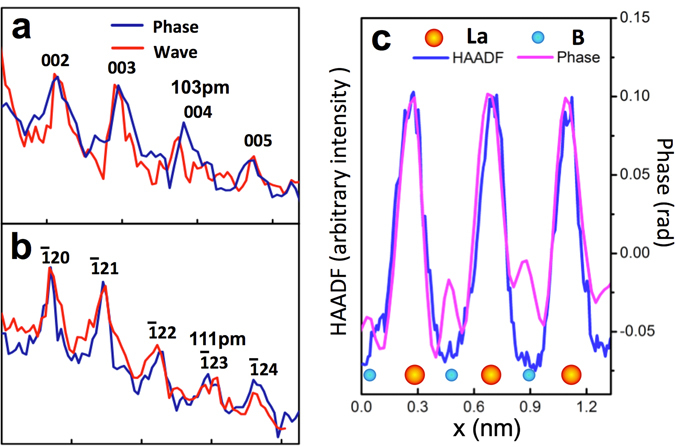
Reciprocal space intensity profiles with a width of 3 pixels extracted from power spectra along the lines L1 (**a**) and L2 (**b**) marked in Fig. 2(c) (**phase** ) and Fig. 2(d) (**complex wave** ), respectively. (**c**) Line profiles with a width of 3 pixels extracted from the reconstructed phase and the HAADF image along the lines marked with green arrows in Fig. 2(a) and (b), respectively.

Combining the ePIE and position refinement algorithms, we have obtained the reconstructed phase (Fig. 2a) from the marked area at the apical region of the LaB_6_ nanoparticle shown in Fig. 1b. The positions of all La atom columns are clearly resolved and agree with the known crystal structure of LaB_6_ in a <210> orientation. The reconstructed phase data are consistent with the conventional HAADF STEM image^47–50^ (Fig. 2b) recorded from the same area, although the latter does not show the positions of the light B atoms due to the power law dependence of the HAADF signal on atomic number^47–50^. In addition, this reconstructed object function was used to calculate the diffraction pattern, which matches the experimental one well as shown in Fig. S6. To compare resolution in the reconstructed phase with that in the HAADF image, Fig. 2c–e show power spectra displayed on a logarithmic scale, calculated from the reconstructed phase (Fig. 2a) and the complex wave of the object and the HAADF image (Fig. 2b). Figure 2b shows a (004) reflection corresponding to a spacing of 104 pm and a weaker $(\bar{1}24)$ reflection corresponding to a spacing of 91 pm. In contrast, power spectra calculated from both the phase and the complex wave (Fig. 2c and d) show strong $(\bar{1}23)\,\,$and (004) reflections, and a weaker (005) reflection, corresponding to spacings of 111 pm, 104 pm and 83 pm. Figure 3a and b show intensity profiles extracted from the positions indicted at L1 and L2, respectively in Fig. 2c (**—)** and d (**—**). Both power spectra show clear peaks corresponding to the (004) and $(\bar{1}23)$ reflections, respectively. However, comparison of the power spectra calculated from the phase to that from the complex wave shows that the (005)and $(\bar{1}24)$ reflections in the latter (Fig. 2d) are slightly weaker than those in the former. Since only the complex wave shows linear information transfer this suggests that a small non-linearity in the information transfer is present the power spectrum calculated from the phase. However, given that these reflections are present in the power spectrum calculated from the complex wave this indicates that the resolution of the reconstruction measured at 83 pm is meaningful. This estimated resolution limit is only slightly worse than the angular limit defined by a 23mrad convergence angle demonstrating a resolution in the ptychographic reconstruction, that is only about a factor of two worse than that achievable using current aberration corrected S/TEM^7–9^ instrumentation.

Insets to Fig. 2a and b show enlargements of the phase of the ptychographic reconstruction and the corresponding HAADF image from the region indicated in Fig. 2a and b, respectively. Projected along a <210> direction, the LaB_6_ structure (top-right inset to Fig. 2a and b) shows individual columns in projection with either sole La or B occupancy. Since the intensity in STEM-HAADF imaging increases with atomic number^7^ as *Z* ^1.7^ the ratio of the STEM-HAADF intensity between B (*Z*_B_ = 5) and La (*Z*_La_ = 57) atoms is approximately 1.5%, explaining why low *Z* chemical elements such as B are not resolved in the presence of heavy elements such as La (inset to Fig. 2b) in this imaging mode. In contrast, the reconstructed phase provides much higher sensitivity to light elements and in the enlarged phase (inset to Fig. 2a) it is clear that additional contrast is visible between the La columns corresponding to four closely separated B atoms in projection along a <210> direction. The elongated shape of these columns is also consistent with the B atom configuration in the atomic model of LaB_6_. Figure 3c shows line profiles (3 pixels wide) extracted from the both the reconstructed phase and HAADF images along the directions of columns containing only La atoms and B (as indicated in Fig. 2a and b). These clearly show contrast from the B atom columns in the reconstructed phase with a contrast ratio between B and La between 20–30%, as compared to zero contrast at the B sites in the HAADF image. This data provides the first experimental demonstration of a ptychographic reconstruction in which structural information from light elements located in proximity to heavy elements is recovered at atomic resolution with high phase sensitivity.

To confirm the interpretation of these experimental results, we have carried out a ptychographic reconstruction from an array of simulated diffraction patterns for a <210> oriented LaB_6_ crystal with a thickness of 10 nm, corresponding to our estimated sample thickness using the multislice method^51^ (for details of the calculation see SI) together with a HAADF image simulation. These simulations are shown in the bottom-right insets to Fig. 2a and b, respectively. It is evident that the B columns are resolved in the ptychographic phase and their locations match those expected for the LaB_6_ structure projected along a <210> direction. The B columns in close proximity to the La columns were not resolved in the current reconstruction as they are located too close to the La columns to be distinguished from the adjacent La columns at the achievable resolution of 80 pm (see also Fig. S8). As expected, simulated HAADF images confirm that the rows of B atom columns are not visible in this imaging mode.

**Figure 4:**
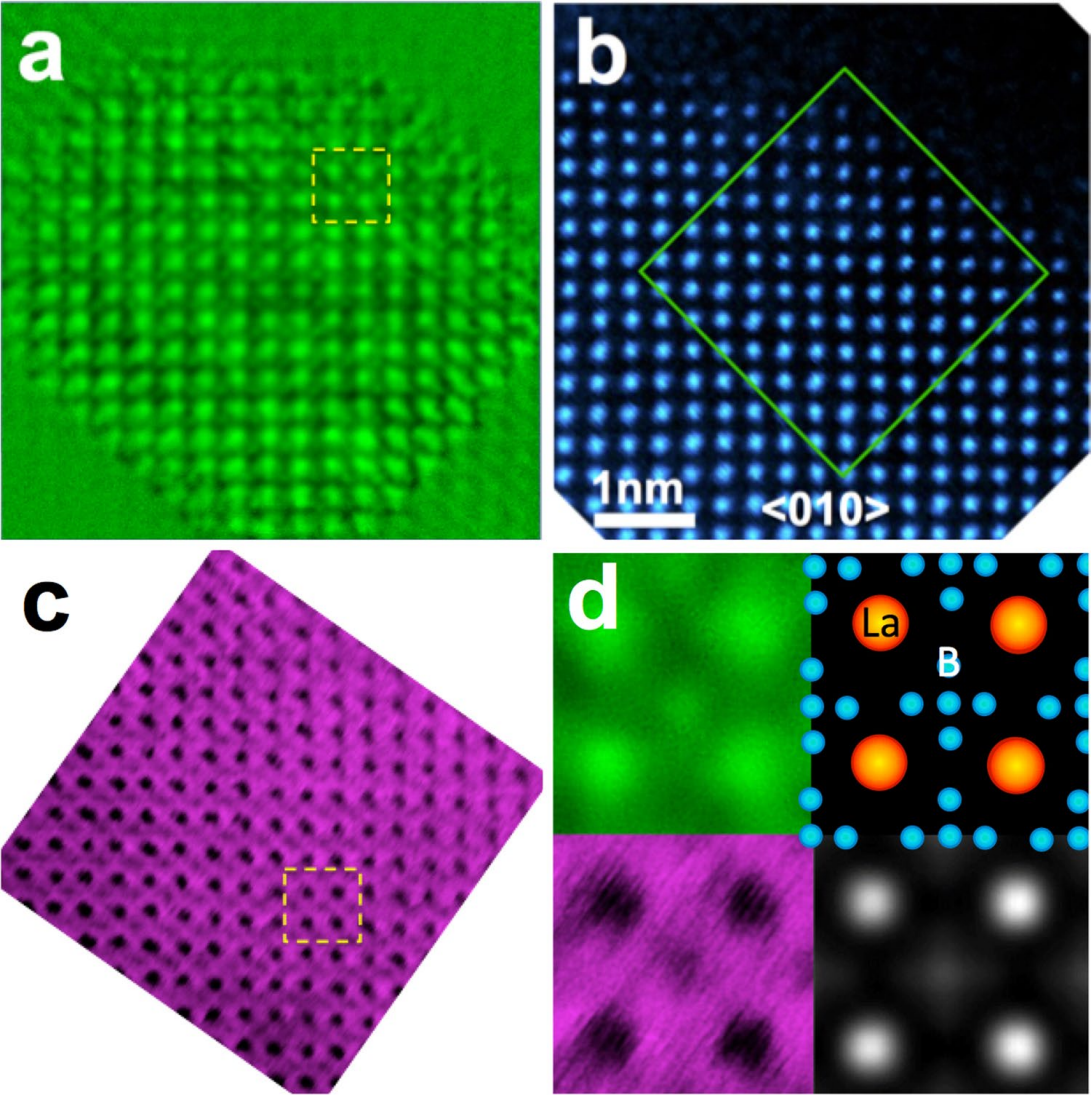
(**a**) Phase of the ptychographic reconstruction for a <010> orientation of a LaB_6_ nanoparticle (total dose 0.94 × 10^8^ e^−^nm^−2^). (**b**) HAADF image from the same region. (**c**) ABF image from a similar nanoparticle to that shown in (**a**) and (**b**) (total dose 2.1 × 10^8^ e^−^nm^−2^). (**d**) Enlarged images (Top-left) and (Bottom-left) extracted from the regions indicated with yellow dashed squares in (**a**) and (**c**), respectively together with projected atomic models of the LaB_6_ structure along a <010> direction (Top-right) and the phase reconstructed from simulated diffraction patterns (Bottom-right).

We have also carried out an additional ptychographic phase retrieval for a smaller crystal region within the same nanoparticle in a <010> orientation using *df*_<010>_  = 65 nm for a 9 × 9 array of diffraction patterns. Figure 4a–c show respectively the reconstructed phase and HAADF and ABF images of the nanoparticle (with the ABF image and its corresponding power spectrum also shown in Fig. S9). In this projection, HAADF imaging only shows contrast from La columns. Figure 4d shows the reconstructed phase (Top-left) and ABF image (Bottom-left) enlarged from the indicated region in Fig. 4a and c, respectively, both of which show a similar additional contrast between the La columns corresponding to the five boron columns in the LaB_6_ structure projected along a <010> projection consistent with the atomic model (Top-right inset to Fig. 4d). This reconstructed phase agrees with a simulation calculated at 80 pm resolution (Bottom-right inset to Fig. 4d, with details of the simulation conditions given in SI and see also Fig. S10). This comparison with ABF imaging shows that the phase reconstructed using ptychography also has sufficient sensitivity to visualize light and heavy atoms simultaneously at comparable electron dose (Table S1 and Fig. S9).

## Conclusions

We have demonstrated that ptychographic reconstruction, including a position-refining algorithm can yield an experimental reconstructed phase with high phase sensitivity at atomic resolution sufficient to provide accurate structural information for both light and heavy atoms in a hexaboride nanoparticle. This type of phase contrast data is potentially capable of mapping local electromagnetic fields with greater sensitivity, which suggests future potential applications of this technique for studies of magnetic and ferroelectric materials^52^. The experimental geometry required for ptychography is compatible with that used for aberration corrected STEM imaging and the required diffraction data set can be readily acquired from the same sample area after or before HAADF STEM images or spectroscopic maps are recorded by altering the objective lens defocus. In this way high-resolution structural data and phase sensitive information can be recovered and correlated to the complementary information provided by other STEM imaging modes. Moreover, refinement of both probe and object functions within the ePIE algorithm makes this approach relatively robust to residual aberrations in the probe. In future, the use of probe position refinement as reported here together with low noise detectors may enable ptychographic reconstruction at lower doses than those reported to date^33^ and comparable to those recently reported for reconstruction from focal series data^53^. There are also potential methods which have been demonstrated in the optical regime by which ptychography can overcome the resolution limits set by partial coherence to recover information beyond the axial information limit^54^. Ultimately, the quantitative phase maps obtained could be used as part of three-dimensional structural characterization^26, 27, 30^. Using a suitably shaped probe, this technique also provides depth sectioning capability^55, 56^ similar to that exploited in confocal imaging^57^ and where the effects of dynamical scattering are accounted for in the phasing algorithm.

## Methods

### Materials

The studies reported used commercially available lanthanum hexaboride powder (Sigma-Aldrich). The powder was ground in a mortar, suspended in isopropanol, ultrasonicated for 10 minutes and then dropcast directly onto holey carbon coated copper TEM grids.

### Experimental Configuration

Data was recorded using an aberration corrected STEM instrument operated at 300 kV with a Schottky field emission source. Figure 1a shows a schematic diagram of the optical configuration used. A probe-forming convergence semi-angle of 23mrad was used and the sample was placed at a distance, *df* above the focal point as shown in Fig. 1a. In this geometry, the overlap between adjacent probe positions were ~85 and 80% for respectively the *df*_<210>_ and *df*_<010>_ experiments, sufficient to fulfill the ptychographic sampling requirement (See SI) for the probe size, scan step and detector pixel size used^58, 59^. At each probe position, a diffraction pattern in reciprocal plane was recorded on a Gatan Ultrascan 1000 CCD camera (with a 16-bit dynamic range and 2048 × 2048 pixels on a 14 μm pitch), from which the central 1k × 1k region was extracted and subsequently used in the ePIE reconstruction. Typical entire arrays of diffraction patterns are shown in Figs S1 and S2.

### Reconstruction

The iterative method for reconstruction used the ePIE algorithm^28^ with a translation position determination algorithm^37^ as summarized below.

The iterative solution in the ePIE algorithm starts with initial estimates of the object, *O*_0_(*r, s*_*j*_) and probe, *P*_0_ (*r*) functions, where *r* is a coordinate in the object plane and *s*_*j*_ is the *j* ^*th*^ object translation shift. An initial condition *O*_0_(*r*, *s*_*j*_) = 1 is assumed and *P*_0_(*r*) is estimated from the known defocus (See SI). The intensity of the diffraction patterns recorded in the far field is defined by *I*_*j*_ (*u*), where *u* is a reciprocal space coordinate.

We subsequently form an estimate of the specimen exit wave function as:

1 $${\psi }_{g}^{j}(r)={P}_{0}(r){O}_{0}(r,{s}_{j})$$

and propagate this exit wave to the diffraction plane *via* a Fourier transform, denoted as ψ_*g*_ (*u*). The modulus of ψ_*g*_ (*u*) is now replaced with the square-root of *I*_*j*_ (*u*) and its phase is preserved, giving:

2 $${{\rm{\Psi }}}_{c}^{j}(u)=\frac{{{\rm{\Psi }}}_{g}^{j}(u)}{|{{\rm{\Psi }}}_{g}^{j}(u)|}\sqrt{{I}_{j}(u)}$$

Transforming back to the object plane gives a revised exit wave ${\psi }_{c}^{j}(r)$ at this position.

An updated estimate of the object function is subsequently computed as:

3 $${O}_{n+1}(r,{s}_{j})={O}_{n}(r,{s}_{j})+{\alpha }_{1}\frac{{P}_{n}^{\ast }(r)}{{|{P}_{n}(r)|}_{max}^{2}}[{\psi }_{c}^{j}(r)-{\psi }_{g}^{j}(r)]$$

where *α*_1_ takes values within [0, 1.5], a range empirically found to give good convergence of this algorithm^39^. In this work *α* = 1 was used and * denotes the complex conjugate and *max* is the maximum value of the function.

An updated estimate of the probe function can now be defined as:

4 $${P}_{n+1}(r)={P}_{n}(r)+{\alpha }_{2}\frac{{O}_{n}^{\ast }(r,{s}_{j})}{{|{O}_{n}(r,{s}_{j})|}_{max}^{2}}[{\psi }_{c}^{j}(r)-{\psi }_{g}^{j}(r)]$$

with *α*_2_ = 1 used in this work.

A similarly updated estimate of the object translation shift is subsequently calculated as:

5 $${s}_{j,n+1}={s}_{j,n}+\beta {e}_{j,n}$$

where, *e*_*j*,__*n*_ is the relative shift error between *O*_n+1_(*r*, *s*_*j*_) and *O*_n_(*r*, *s*_*j*_) and the parameter *β* amplifies the shift position error controlling the rate of refinement.

The parameter *e*_*j*,__*n*_ is calculated by cross correlation of *O*_n+1_(*r*, *s*_*j*_) with *O*_n_(*r*, *s*_*j*_) and an automatic adjustment of *β* was used in the present work^37^. The above are then repeated for the next object position *s*_*j*+1_ until all the probe positions have reached a single iteration of the ePIE algorithm. This procedure was run for 300 iterations for a single reconstruction. Details of the procedures underlying ePIE algorithm and the position determination algorithm have been reported elsewhere^28, 37^ and an example of a resultant position refinement is shown in Fig. S3.

## Electronic supplementary material

SUPPLEMENTARY INFO
