# Supplementary material for: Electron Ptychographic Diffractive Imaging of Boron Atoms in LaB6 Crystals
Source: Sci Rep. 2017 Jun 6;7:2857. doi: 10.1038/s41598-017-02778-x (PMC5460146; doi:10.1038/s41598-017-02778-x)
Supplement: Supplementary file 1 — SUPPLEMENTARY INFO [file 41598_2017_2778_MOESM1_ESM.pdf]

Supplementary information for:

Electron Ptychographic Diffractive Imaging of Boron Atoms in LaB_6 Crystals

Peng Wang[1*], Fucai Zhang[2,3,4*], Si Gao[1], Mian Zhang[1]and Angus I. Kirkland[4,5,6]

[1]*National Laboratory of Solid State Microstructures, College of Engineering and Applied Sciences and Collaborative Innovation Center of Advanced Microstructures, Nanjing University, Nanjing 210093, People's Republic of China.*

[2]*Department of Electrical and Electronic Engineering, Southern University of Science and Technology, Shenzhen 518055, China.*

[3]*London Centre for Nanotechnology, London WC1H 0AH, UK.*

[4]*Research Complex at Harwell, Harwell Oxford Campus, Didcot OX11 0FA, UK.*

[5]*Department of Materials, University of Oxford, Parks Road, Oxford OX1 3PH, UK.*

[6]*Electron Physical Sciences Imaging Centre, Diamond Lightsource Ltd., Diamond House, OX11 0DE, U.K.*

* Correspondence to: wangpeng@nju.edu.cn; zhangfc@sustc.edu.cn

<u>Initial and Reconstructed Probe Functions.</u>

An initial estimate of the probe function $P_0(r)$ is required for ptychographic reconstruction using the ePIE algorithm. To achieve this the experimental amplitude was obtained from the inverse Fourier transform of a Ronchigram taken in the absence of the sample (Fig. S7). The phase of the initial probe function, $P_0(r)$, was calculated using the aberration coefficients measured from the electron-optical microscope alignment[1] immediately before data acquisition. The distances, *df* between the sample and the probe crossover were further refined using knowledge of the inter-atomic spacing between columns of La atoms in <210> and <010> projections. Fig. S4 shows the amplitudes of the initial probe functions (a & c) and reconstructed probe functions (b & d) using the ePIE algorithm at the sample plane for $df_{<210>}$ = 98 nm and $df_{<010>}$ = 65 nm, respectively.

Multislice Simulation of Diffraction Patterns for Ptychography (bottom-right inset in Figs. 2a and 4c).

Simulated phases (bottom-right insets to Figs. 2a and 4c) were reconstructed from diffraction patterns calculated using the multislice method[2] using code due to Kirkland[3]. Model specimens were constructed as <210> and <010> oriented LaB_6 crystals with a thickness of 10nm, respectively. The incident electron energy was 300 keV. The simulated diffraction pattern was calculated on a 1024 x 1024 pixel array with samplings of 0.085 and 0.215 mrad/pixel, respectively. The phases were subsequently recovered using ePIE as shown in Fig. S8 and 10. To evaluate the effects of the experimentally limited resolution, the reconstructed phase was convolved with a Gaussian function with a full width at half maximum (FWHM) equal to the limiting resolution. Fig. S8c and S10c show examples with FWHM = 0.08 nm. This convolution was used for the data presented as bottom-right insets in Figs. 2a and 4c, respectively. For simulation of diffraction patterns, the “frozen phonon” method was included in the multislice simulation and compared to simulations without phonon states includes. Including frozen phone states the calculated intensity of diffraction patterns is, therefore, an incoherent superposition of the images formed for each atomic configuration over the range of atomic positions given by the Debye-Waller factors (0.0926Å and 0.0949Å for La and B atoms, respectively[4]). For these calculations 20 configurations[5] were used to converge to a precision better than 2% in simulating the HAADF image contrast. The value of the phase reconstructed in the simulation with TDS decreases by 10% in comparison to that without TDS, as shown in Fig. S11. No noticeable broadening of the width of the atomic columns is observed.

The Ptychographic Sampling Condition.

In a ptychographic dataset the strict Shannon sampling restriction, required for conventional coherent diffractive imaging, is relaxed[6,7] for each diffraction pattern. We define the ptychographic sampling requirement using the ptychographic sampling ratio as:

$$\hat{S}_{x,y} = \frac{\lambda}{2\Delta\theta \cdot \Delta R} \tag{1}$$

which provides a measure of the extent to which intensity in the ptychographical dataset is oversampled above the requisite minimum $\hat{S}_{x,y} = 1$, in the x-y plane. For this work $\lambda = 0.00197\text{nm}$ corresponding to an incident electron energy of 300kV, $\Delta\theta = 0.085\text{mrad}$ the angle subtended at the specimen by a detector pixel in the experimental configuration used and ΔR is the distance between probe positions in the object plane. For the crystal orientations reported, ΔR are nominally equal to 0.48 nm and 0.45 nm and $\hat{S}_{x,y}$ for (210) and (010) data are 24 and 10, respectively. Hence, the ptychographical dataset used here is oversampled beyond the minimum sampling requirement[4].

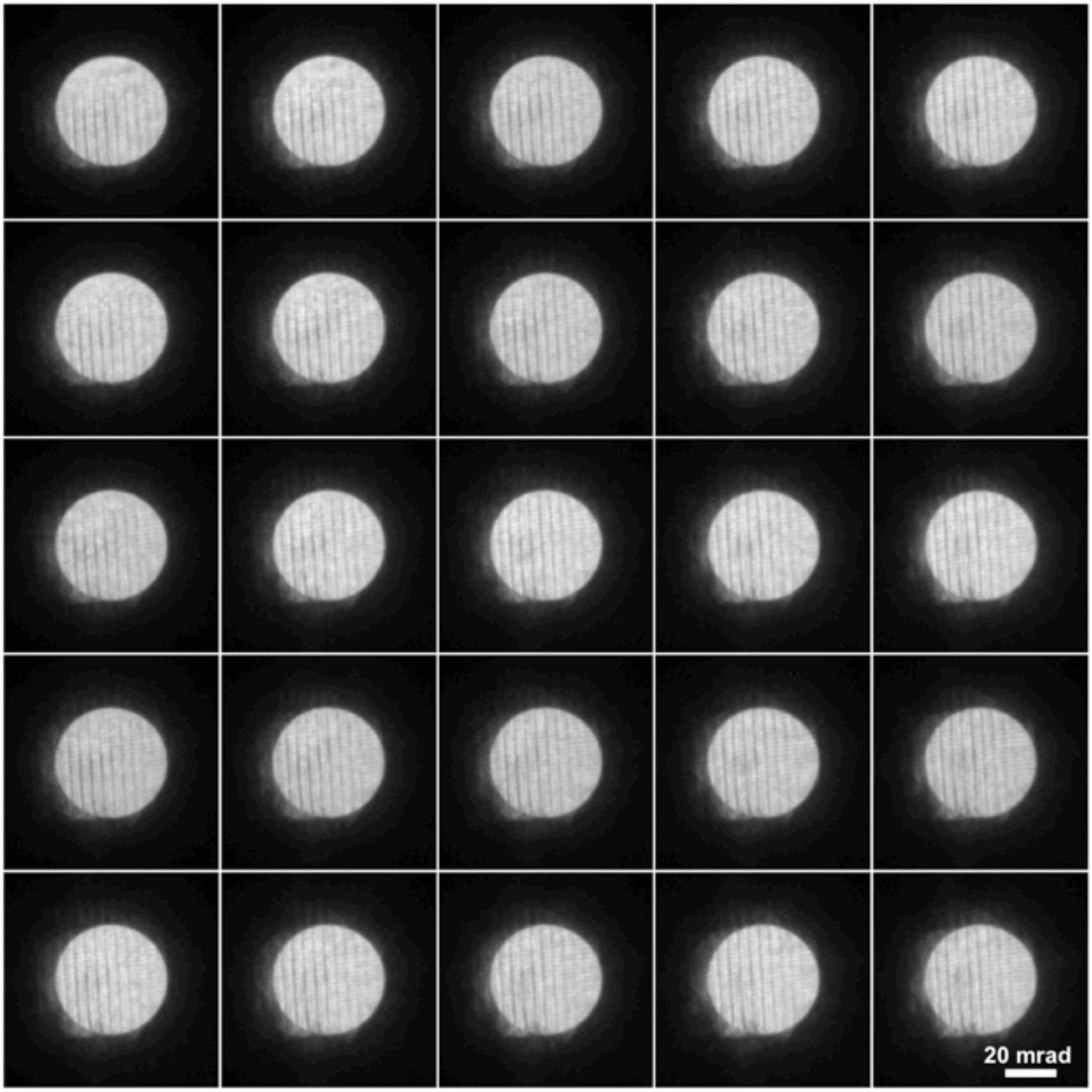

Figure S1. 5x5 array of diffraction patterns recorded with $df_{<210>}$ = 98 nm from the area of specimen indicated with a green box in Fig. 1b.

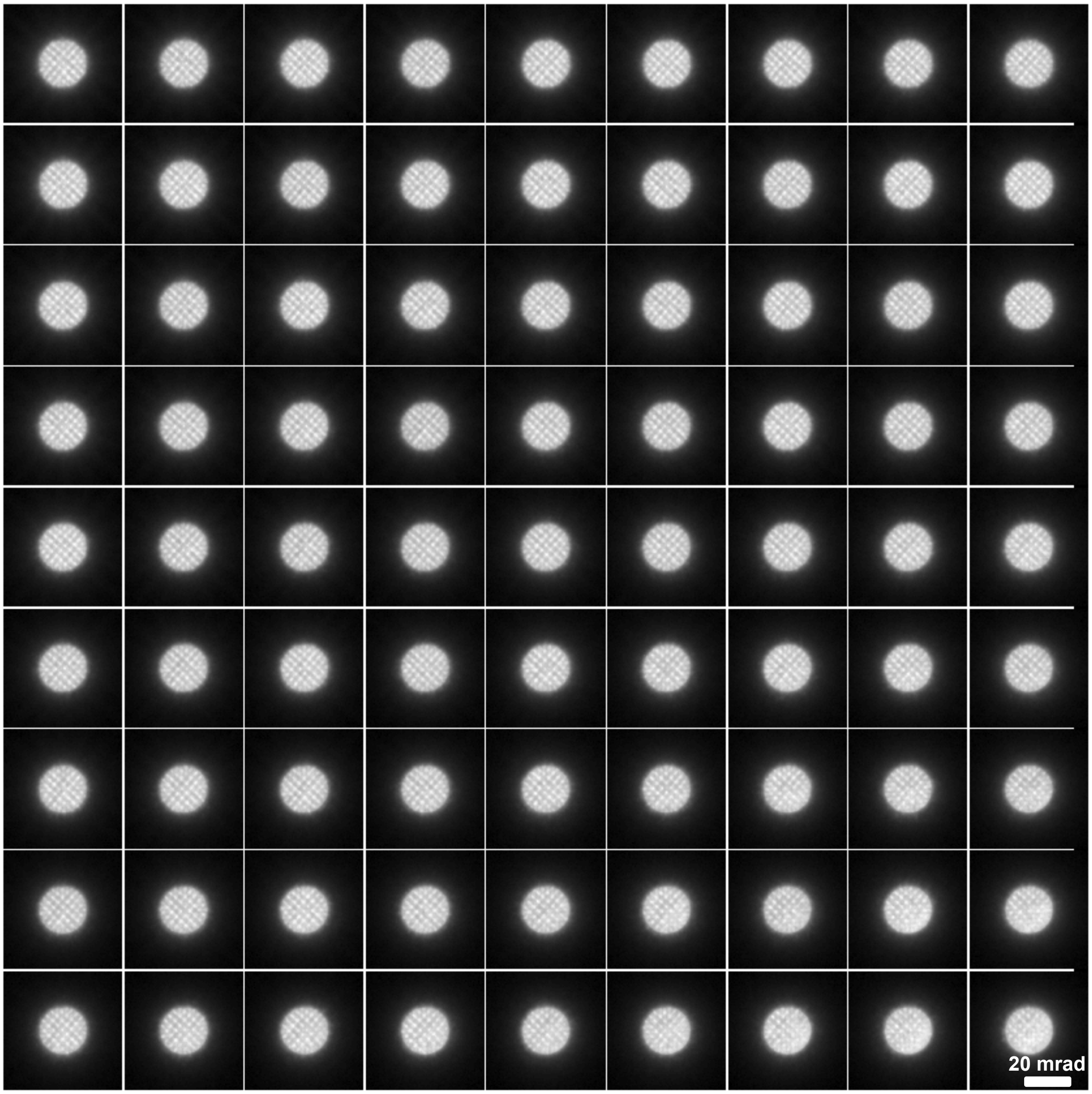

Figure S2. 9x9 array of diffraction patterns recorded with $df_{<010>}$ = 65 nm from the area of specimen indicated with a green box in Fig. 4b.

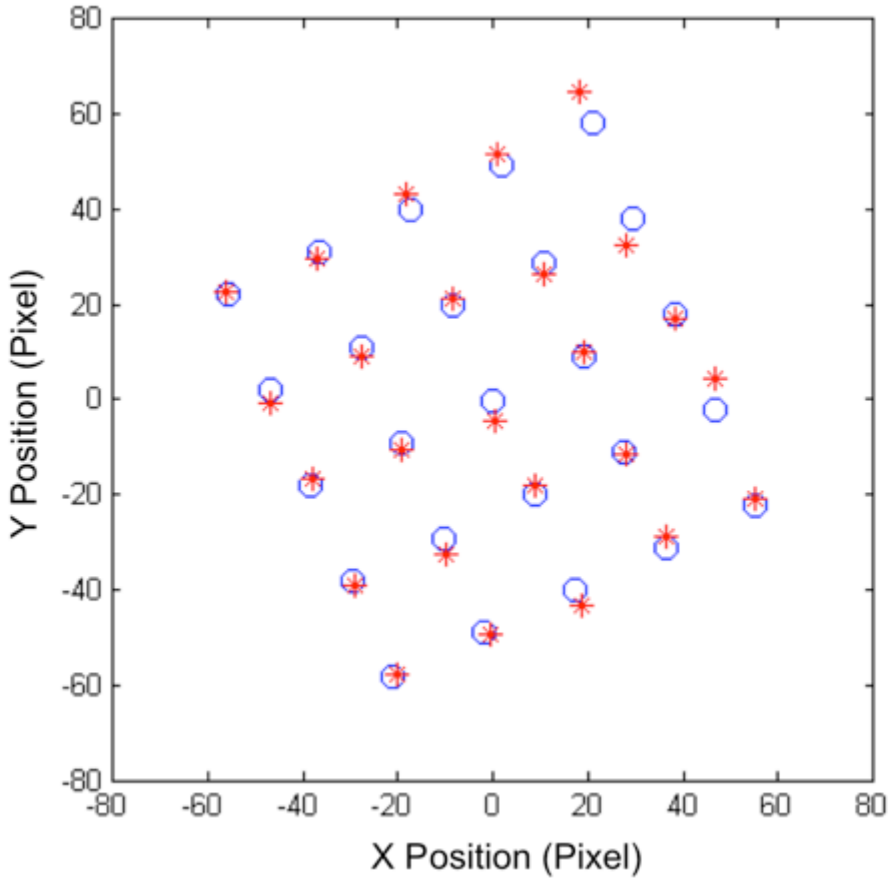

Figure S3. Comparison of positions between nominal X-Y scans (o) and retrieved probe positions (*) used in the reconstruction of region as shown in Fig. 2a. Deviations are due to experimental errors arising from inaccuracies in the position of the illumination largely due to hysteresis in the microscope shift coils.

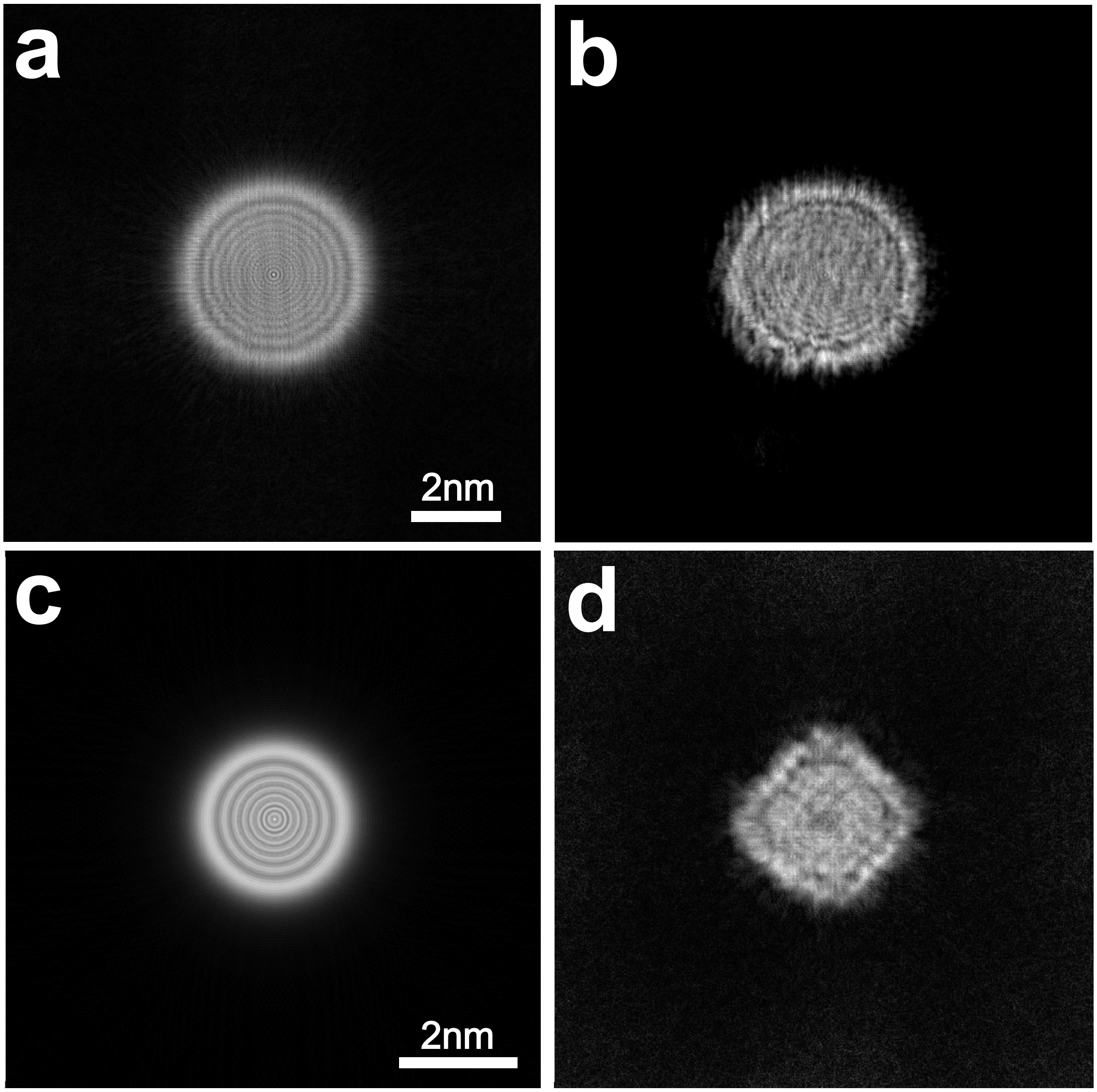

Figure S4. Estimated and reconstructed probe functions using the ePIE algorithm. Moduli of estimated (a, c) and reconstructed (b, d) probe functions with $df_{<210>}$ = 98 nm and $df_{<010>}$ = 65 nm using the ePIE algorithm, respectively.

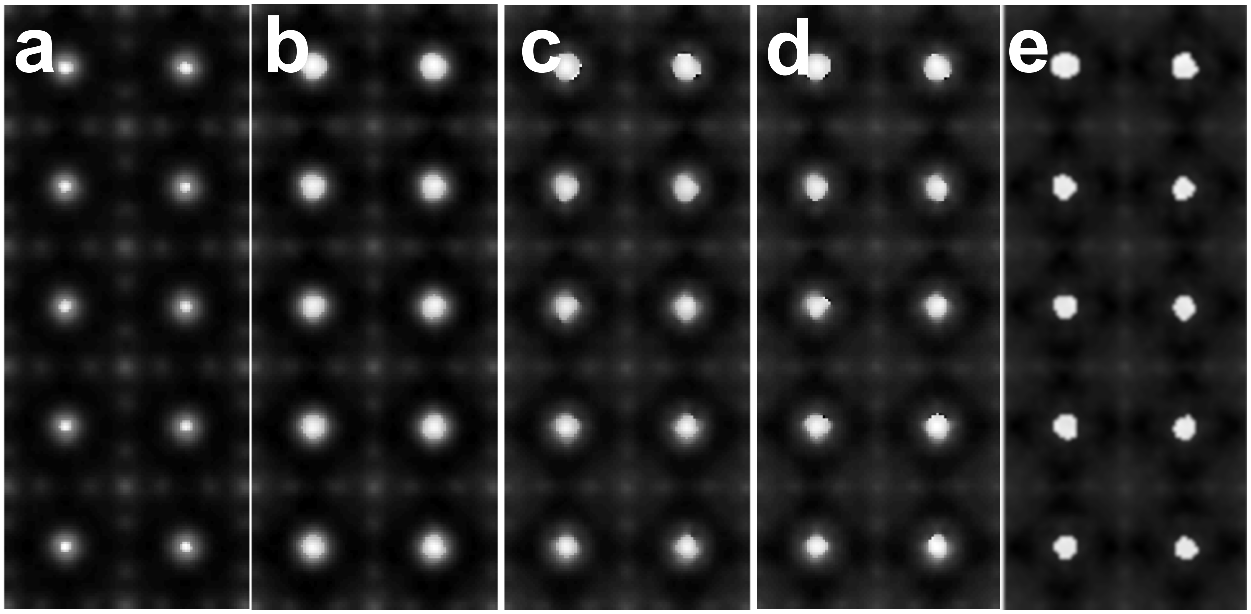

Figure S5. Phase reconstructed from diffraction patterns calculated using the multislice method[3] with $df_{<010>}$ = 65 nm for a <010> oriented LaB_6 crystal with a ±50% variation in a 10nm crystal thickness (a) 5nm, (b) 8.3 nm, (c) 10nm, (d) 11.7nm and (e) 15nm, respectively. Phases match the experimental data shown in Fig. 4a across the full range of thicknesses. These simulations show that the method described is robust to variations in thickness across a range of thicknesses within the boundaries of possible errors in thickness measurement using the EELS log-ratio.

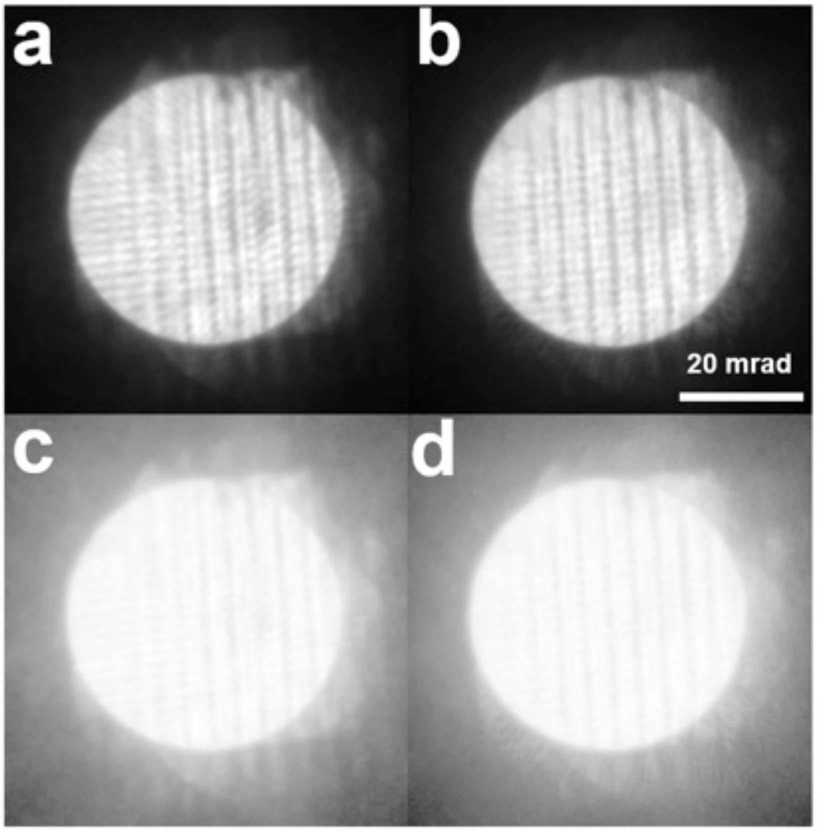

Figure S6. Experimental and Reconstructed diffraction patterns (a) Experimental diffraction patterns from the sample with $df_{<210>} = 98$ nm and (c) displayed on a log-scale. Strong bright-field intensity is visible inside the central disk, (the Gabor hologram or Ronchigram). (b) Diffraction pattern calculated from the ptychographically reconstructed object function and (d) displayed on a log-scale.

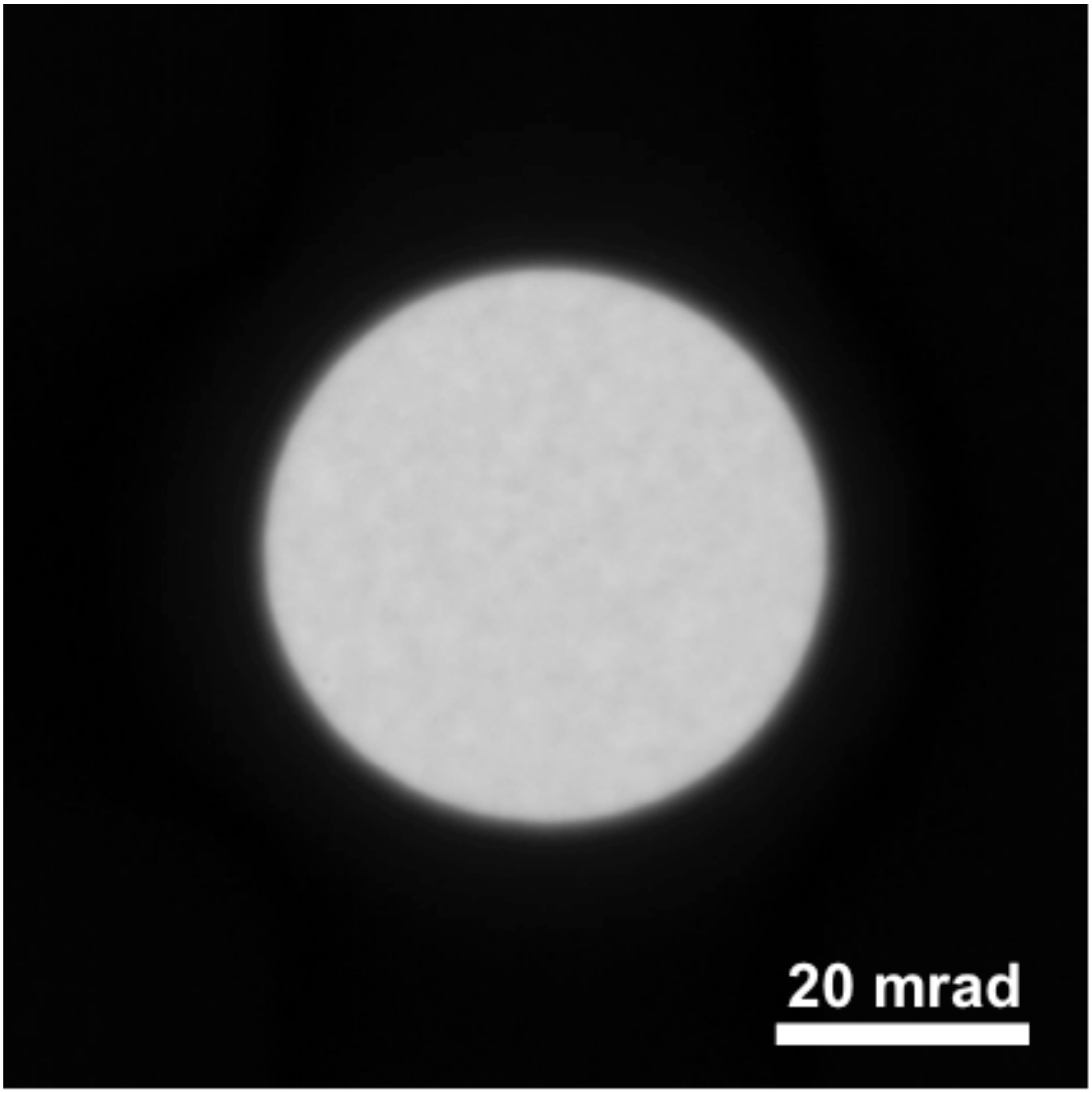

Figure S7. Diffraction pattern recorded in the absence of the sample.

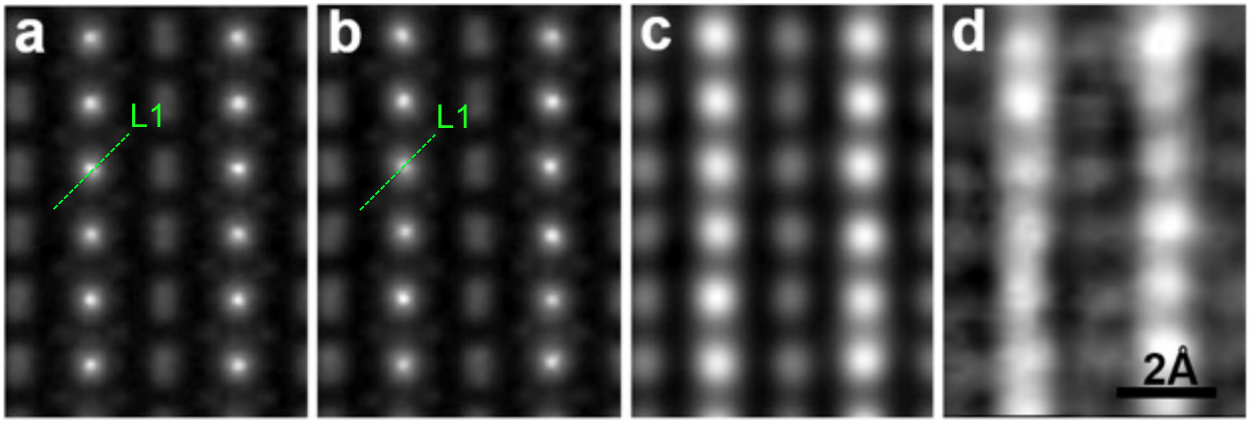

Figure S8. Phase reconstructed from diffraction patterns simulated using the multislice method[3] and experimentally data. Ptychographic reconstructed phase from simulated diffraction patterns with $df_{<210>}$ = 98 nm for a <210> oriented LaB_6 crystal with a thickness of 10 nm (a) without and (b) including TDS using the frozen phonon model. The standard deviation of La and B atoms used were 0.0926Å and 0.0949Å, respectively[4]. (c) Phase calculated from (b) following convolution with a Gaussian function with a full width at half maximum (FWHM) of 0.8 nm shown as the bottom-right inset to Fig. 2a. (d) Phase reconstructed from the experimental diffraction patterns (Top-right inset to Fig. 2a).

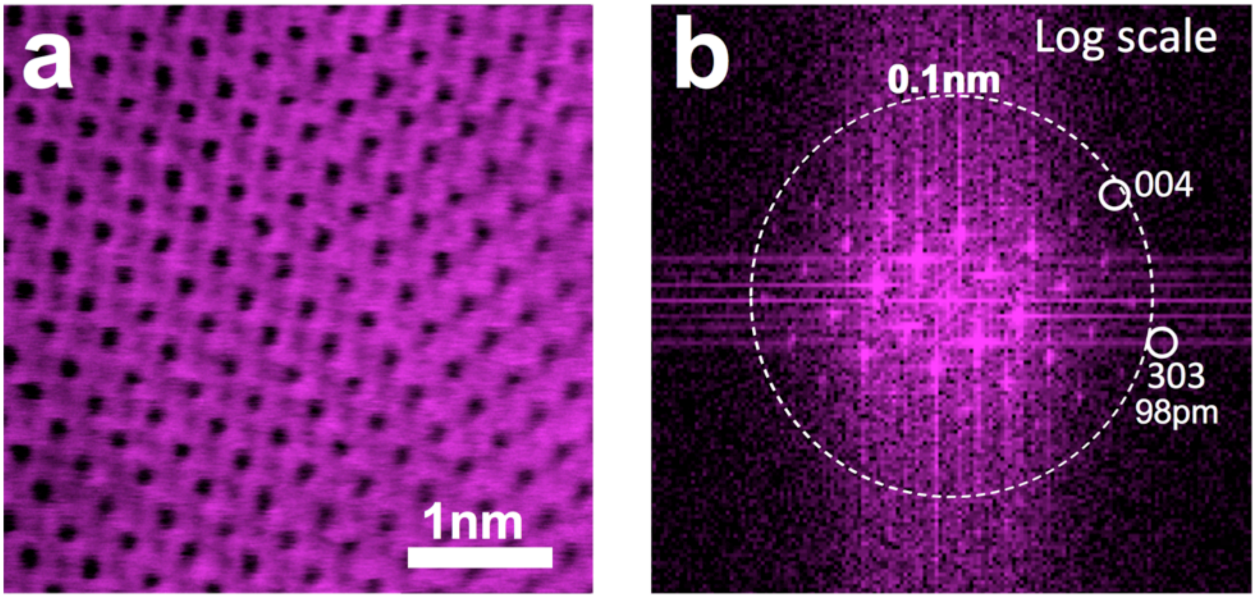

Figure S9. (a) ABF image of a <010> oriented LaB_6 crystal. (b) Power spectra of (a) displayed on a logarithmic intensity scale. Circles indicate (004) and (303) reflections of the LaB_6 lattice corresponding to spacings of 104pm and 98pm. The dotted circle indicates a 100pm resolution limit. The ABF STEM image is affected by the presence of residual aberrations due to the drift of lower order aberrations during operation, which lowers the achievable resolution than the information limit of 0.07 nm. This is also a potential advantage of ptychography, which is less sensitive to the presence of residual aberrations as both the probe and object function can be fitted.

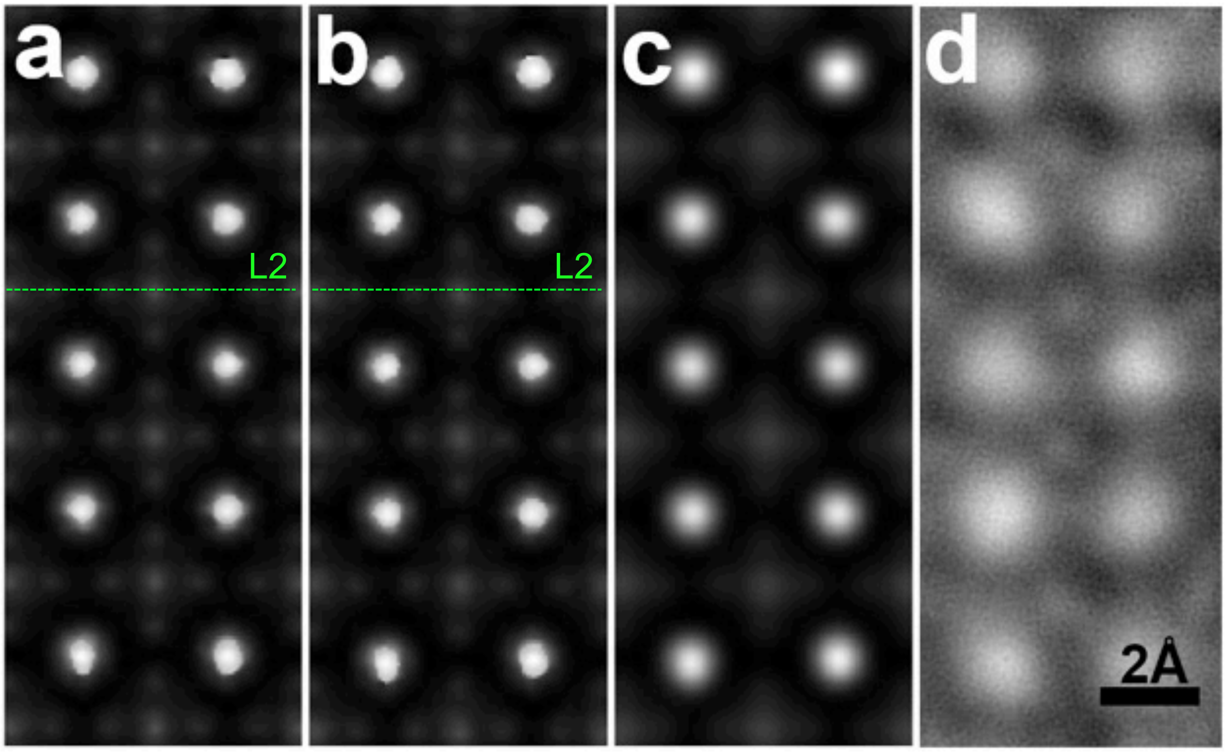

Figure S10. Phase reconstructed from diffraction patterns simulated using the multislice method[3] compared to experimental data. (a) Ptychographic reconstructed phase from simulated diffraction patterns with $df_{<010>}$ = 65 nm for a <010> oriented LaB_6 crystal with a thickness of 10 nm (a) without and (b) including TDS using the frozen phonon model. The standard deviation of La and B atoms are 0.0926Å and 0.0949Å, respectively[4]. (c) Phase calculated from (b) following convolution with a Gaussian function with a full width at half maximum (FWHM) of 0.8 nm shown as the bottom-right inset to Fig. 4d. (d) Phase reconstructed from the experimental diffraction patterns (Top-right inset to Fig. 4d).

	ABF	Ptychography
Total Data Size (pixel)	1024x1024	1200 x 1200 x 9 x 9
Total Time (s)	12.6	0.1 x 9 x 9
Pixel Size (nm)	0.0053	0.0088
Scan Area (nm^2)	29.5	42.9
Current (pA)	80	80
Dose (e/nm^2)	2.1×10^8	0.94×10^8

Table S1. Experimental conditions used to record experimental ABF and ptychographic data as described in the text.

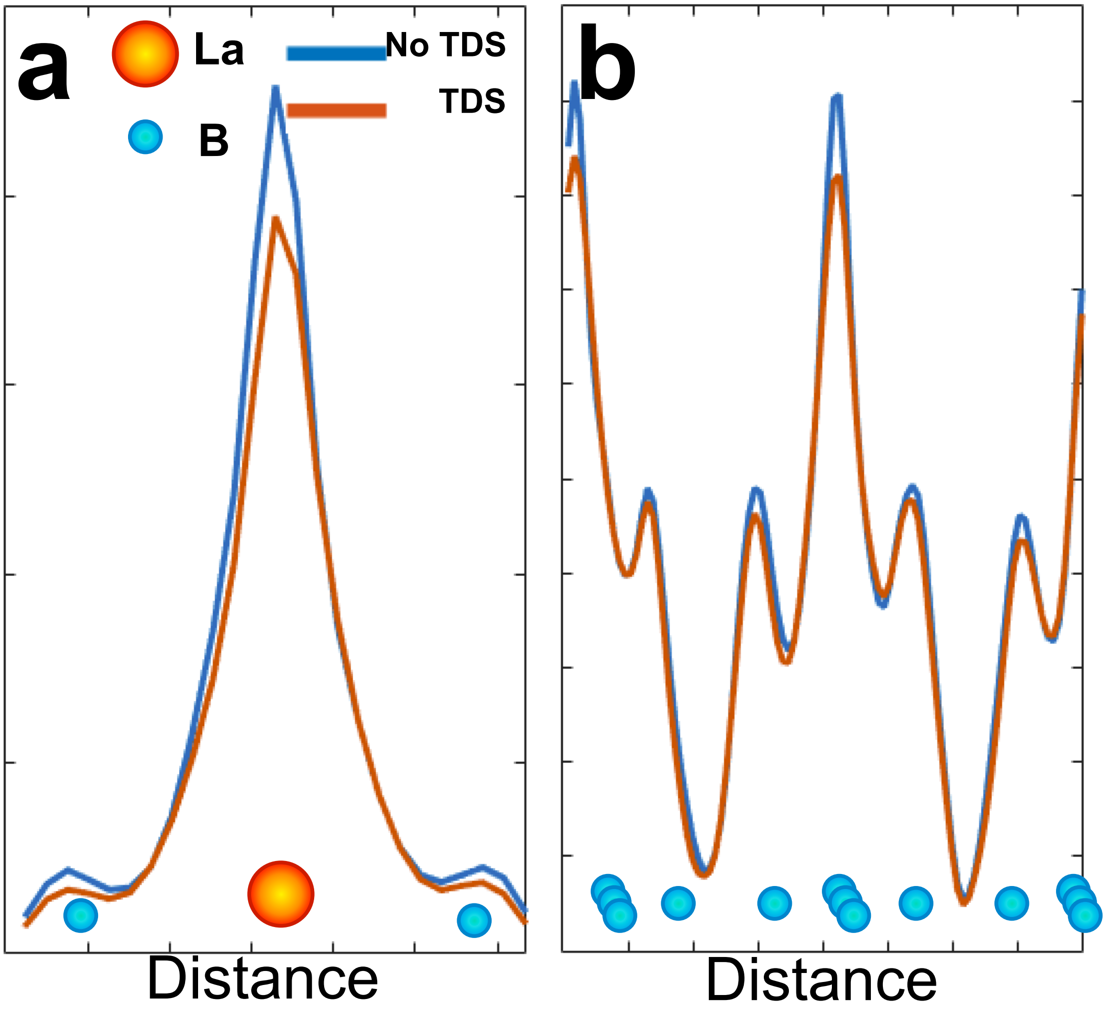

Figure S11 Line profiles with a width of 3 pixels extracted from the simulations with (—) and without (—) TDS along the positions marked with green dotted lines, (**a**) L1 in Fig. S8 **a** & **b** and (**b**) L2 in Fig. S10 **a** & **b**, respectively. The value of the phase reconstructed in the simulation with TDS decreases by 10% in comparison to that without TDS. No noticeable broadening of the width of the atomic columns is observed.

Supplementary References.

1 Sawada, H. *et al.* Measurement method of aberration from Ronchigram by autocorrelation function. *Ultramicroscopy* **108**, 1467-1475 (2008).

2 Cowley, J. M. & Moodie, A. F. The scattering of electrons by atoms and crystals. I. A new theoretical approach. *Acta Crystallographica* **10**, 609-619 (1957).

3 Kirkland, E. J. *Advanced Computing in Electron Microscopy*. (Plenum, 1998).

4 Pecharsky, V. K. & Zavalij, P. Y. *Fundamentals of powder diffraction and structural characterization of materials*. Vol. 69 (Springer, 2009).

5 Loane, R. F., Xu, P. & Silcox, J. Thermal vibrations in convergent-beam electron diffraction. *Acta Crystallographica Section A* **47**, 267-278 (1991).

6 Edo, T. B. *et al.* Sampling in x-ray ptychography. *Physical Review A* **87**, 053850 (2013).

7 Batey, D. J. *et al.* Reciprocal-space up-sampling from real-space oversampling in x-ray ptychography. *Physical Review A* **89**, 043812 (2014).
